# Effect of Daily Egg Ingestion with Thai Food on Serum Lipids in Hyperlipidemic Adults

**DOI:** 10.5402/2013/580213

**Published:** 2013-10-28

**Authors:** Supanee Putadechakum, Pariya Phanachet, Varapat Pakpeankitwattana, Theerawut Klangjareonchai, Chulaporn Roongpisuthipong

**Affiliations:** ^1^Research Center and Division of Nutrition and Biochemical Medicine, Department of Medicine, Faculty of Medicine, Ramathibodi Hospital, Mahidol University, Bangkok 10400, Thailand; ^2^Division of Nutrition and Biochemical Medicine, Department of Medicine, Faculty of Medicine, Ramathibodi Hospital, Mahidol University, Bangkok 10400, Thailand; ^3^Department of Food Chemistry, Faculty of Pharmacy, Mahidol University, Bangkok 10400, Thailand; ^4^Division of Endocrinology and Metabolism, Department of Medicine, Faculty of Medicine, Ramathibodi Hospital, Mahidol University, Bangkok 10400, Thailand; ^5^Bumrungrad International Hospital, Bangkok 10110, Thailand

## Abstract

Thai food is one of the healthiest foods. In fact, several Thai dishes, such as Tom Yum soup, are currently under scientific study for their incredible health benefits. Limited data are available on the effects of egg consumption with Thai food in hyperlipidemic patients. To assess the effects of daily egg consumption with Thai food, which is known as low fat diet, on serum lipids profiles in hyperlipidemic subjects without medication treatment, the randomized crossover trial of 71 hyperlipidemic adults (8 men, 63 women) were randomly to one of the two sequences of one and three eggs/day for 4 weeks. Each treatment was separated by a four-week washout period (egg-free). Our data indicated that one or three eggs/day consumption were significantly increases total serum cholesterol (221.54 ± 42.54 and 225.31 ± 45.06 versus 211.57 ± 39.98 mg/dL) and LDL-C levels (141.38 ± 38.23 and 145.48 ± 39.33 versus 133.44 ± 34.52 mg/dL) as compared to egg-free period. No significant change of serum TG, HDL-C, TC/HDL-C, and LDL-C/HDL-C levels was observed after 1 or 3 eggs consumption daily in this study.

## 1. Introduction

Half of the world's population is living in Asia they had their own culture of eating. Rice is a main food with different kinds of plant including vegetables, bean, legume, and herbs. The traditional Asian diet is one of the health model diets because of low incidence of chronic diseases in Asian countries [1]. Thai food is one of the Asian foods, of good taste, and contains various healthy vegetables, herbs, and spices along with rice and noodles but is less in fat. Thai food does not contain big meat chunks of large animals; on the other hand, they are fortified and shredded with various herbs and healthy vegetables [2]. Herbs and spices in Thai foods may boost metabolism and fight inflammation. Thai food is one of the most intrinsically healthy foods in the world [3]. Thai people consumed lower egg than those in other countries which was only 130 eggs per capita per year whereas 230 and 350 in Hong Kong and Japan, respectively. From 2012, Thai people were encouraged to consume more eggs due to low-cost but high-quality protein of eggs [4]. Data from Thai National Nutrition Survey in 1986 revealed that average fat intake in Thai people was 21.8% of total calories consumption [5]. Fat consumption in Thai people resemble to that of the NCEP step I diet (≤30% of total energy as fat). NCEP step I diet consists of ≤30% of total energy as fat, ≤10% of energy as SFA (saturated fatty acids), and ≤300 mg dietary cholesterol/day [6, 7]. Limited data are available on the effects of egg consumption with Thai food in hyperlipidemic patients. So, this study tries to assess the effects of daily egg consumption with Thai food, which is known as low fat diet, on serum lipid profiles in hyperlipidemic subjects without medication.

## 2. Materials and Methods

### 2.1. Subjects

Eighty adults diagnosed as hyperlipidemic participants (total serum cholesterol (TC) > 200 mg/dL; LDL-cholesterol (LDL-C) ≥ 130 mg/dL), without medication, were recruited. The participants who have been diagnosed as having egg allergy, hypothyroidism, coronary heart disease, liver or kidney problems as well as the use of hormone replacement therapy were excluded from the study. The study was a randomized crossover design trial. Participants were randomly assigned to one of the two arms of one egg per day (EGG 1) and 3 eggs per day (EGG 3) for 4 weeks. Each study arm assignment was separated by a four-week washout period (egg-free, EGG 0). Subjects were not provided any other foods apart from eggs for their additional diet. They were asked to maintain their normal routine dietary intake and physical activity during the whole period of the study. Measurement of body weight, body mass index, body composition by bioelectrical impedance, blood pressure, serum lipid profiles, 3-day dietary record, and daily egg consumption were performed and collected every 4-week interval except for height which was done only once at baseline. The biochemical tests including plasma glucose, renal and liver function tests, and haematological parameters were measured before and after the study. 

The study protocol had been reviewed and approved by the Ethical Committee on Human Rights related to Researches Involving Human Subjects of Ramathibodi Hospital, Mahidol University. All participants were informed in detail about the study, and written informed consents were obtained.

### 2.2. Dietary Assessment

Dietary assessment for macronutrients was determined by 3-day, 24-hour dietary records. They were required to maintain the food records for 2 weekdays and 1 weekend day. The interviewers collected the data and estimated the serving portions with the use of household measuring cups, spoons, and ruler to assist the subjects in the recall of eaten foods. The quantitative calculation of each macronutrient was analyzed. During the study, the subjects were asked to check on a daily egg record sheet.

### 2.3. Lipid Profile

Serum lipid profile was assessed every 4-week interval, including total serum cholesterol (TC), triglyceride (TG), and high-density lipoprotein cholesterol (HDL-C) which were measured by enzymatic methods (Boehringer Mannheim Corporation, Mannheim, Germany) after 12 hours overnight fast. Low-density lipoprotein cholesterol (LDL-C) was calculated from Friedewald's formula [8].

### 2.4. Body Composition

Body composition was determined by bioelectrical impedance method. Body composition was measured in the morning after an overnight fast. Body mass was recorded to the nearest 100 gram on a calibrated digital scale. 

### 2.5. Statistical Analysis

Participant characteristics and serum lipid profile of all subjects were reported by using mean ± standard deviation (SD). Statistical analysis was conducted using SPSS software version 13.0 for windows. All outcome measurements among baseline and each period data were assessed using two-way repeated measures ANOVA. *P* < 0.05 was considered statistically significant. Concerning egg supplement effects, the final results of 2 arms were assessed using paired student's *t*-test. 

## 3. Results

Seventy-one hyperlipidemic subjects, with no medication, participated in this study. Eighty-nine percent of the subjects were female. They were 23 to 75 years old, with a mean age of 50.79 years (Table 1). Five participants dropped out from the study because of unwillingness to consume eggs every day, and four participants dropped out because of poor compliance to 12-week program of the study.

Body weight, BMI, BMR (basal metabolic rate), % Body fat, WHR (waist over hip ratio), % body cell mass, % lean body mass, total body water, and systolic, diastolic BP did not show significant change (Table 1). 

No statistical difference of time sequence effect to consume 1 or 3 eggs was found during the study, the results from the two groups were pooled. Additional consumption of 1 egg (EGG 1) and 3 eggs (EGG 3) per day for 4 weeks increased subjects' serum TC (8.94, 13.54 mg/dL) and LDL-C (7.94, 14.53 mg/dL) levels from washout period (EGG 0) but no significant change of TG, HDL-C, TC/HDL-C and LDL-C/HDL-C ratio from EGG 0 (Table 2). The biochemical tests of plasma glucose, renal and liver function tests, and hematological test had no significant change before and after the study.

## 4. Discussion

The association between dietary cholesterol and coronary events and mortality is generally positive. Whether dietary cholesterol and cardiovascular risk, association between dietary and serum cholesterol are related, these issues are controversial. The results from our study revealed that 1 egg or 3 eggs consumption with Thai food had unfavorable influence on serum TC, LDL-C levels in the study population of hyperlipidemic adults without medication. After 4 wk of EGG 1 and 3 the subjects' serum TC and LDL-C significantly increased from EGG 0 (TC: 221.54 (*P* < 0.034), 225.31 (*P* < 0.006) versus 212.18 mg/dL and LDL-C: 141.38 (*P* < 0.035), 145.48 (*P* < 0.0005) versus 133.67 mg/dL). The study showed no significant change of serum TG, TC/HDL-C, and LDL-C/HDL-C (Table 2). Additional consumption of 1 egg or 3 eggs per day for 4 weeks tended to increase serum HDL-C level although there was no statistic significant difference.

 A clinical trial in healthy postmenopausal women aged 60 years or older had showed that the participants who were classified as hyporesponders to dietary cholesterol had no change in LDL-C and HDL-C after consuming 3 eggs per day for 4 and 12 weeks. On the other hand, the participants who were classified as hyperresponders exhibit increases in both LDL-C and HDL-C with no change in the LDL-C to HDL-C ratio [9]. A study of daily 2 eggs consumption for 6 weeks in 40 hyperlipidemic adults (24 women, 16 men) with serum TC of 244 ± 24 mg/dL had no adverse effect on endothelial function and serum cholesterol or other measures of their lipid profiles when compared to sausage and cheese ingestion [10, 11]. The lipid profile after consuming additional 3 eggs per day in hyperlipidemic adults, who were treated with lipid lowering drugs, showed no change in LDL-C, increase in HDL-C, and decrease in LDL-C/HDL-C ratio [12]. These findings harmonized with a previous study of the effect of egg consumption on patients on HMG-CoA reductase inhibitor which showed significant increases in serum HDL-C in subjects given 2 or 4 eggs daily [13]. The absence of an increase in plasma cholesterol in hyporesponders may be due to their ability to maintain cholesterol homeostasis by decreasing absorption of dietary cholesterol or suppressing endogenous synthesis [14].

 A randomized controlled cross-over study of Chakrabarty et al. [15] was performed on eighteen healthy young volunteers (7 males, 11 females) on a lacto-vegetarian diet. They were given one boiled egg per day for 8 weeks. Their serum TC after 4 weeks of egg consumption were significantly higher than that of the reference values. Furthermore, seven out of 18 subjects had an appreciable elevation of serum TC or LDL-C or both after 8 weeks of egg consumption. Similar results to our data showed increase of serum TC and LDL-C levels in the participants after 4 wk of EGG 1 and EGG 3 ingestion, probably due to high dietary fat intake habit of our participants but no dietary program provided. We even expect that our study population will consume low fat in their Thai food but we did not suggest that they change their eating habit. 

When we compared our study to Techakriengkrai et al. [16], the results showed no significant change of serum TC, LDL-C, TG and HDL-C while they were strict on a cholesterol lowering diet (NCEP step I diet) with 1 and 3 eggs ingestion for 4 wk. The sequence of no egg and 1 egg/d had significant lower serum TC and LDL-C (*P* < 0.05) as compared to those of the baseline which did not take NCEP step I diet which recommends that no more than 30% of total calories be from fat and no more than of 10% of total calories be from saturated fat. In addition, subjects were instructed to consume less than 300 mg of dietary cholesterol per day [17]. NCEP step I diet can decrease total cholesterol and LDL cholesterol by <7–9% [18]. Fat consumption in our study was 34.12–34.94% of total calories which was higher than NCEP step I diet (30%). Thai food contains lots of fibers, carbohydrates, vitamins, and minerals with limited or restricted quantities of animal fats and protein sources as well. Authentic Thai cuisine is extremely healthy since it was derived from the best healthy cooking methods such as stewing, grilling, and baking [4]. Globalization, technologies, and socioeconomic have changed people in living style as well as eating habit due to fast growing era. People consumed more fatty diet than ever. Method, of cooking like frying, deep frying, and stir frying were introduced into preparation procedure of Thai diet by western countries as well as fast food. It is evident that Thai people consumed more fat in a past decade from the Thai National Nutrition Survey in 2003 [19] using a 24 hrs food intake recall; fat intake in Thai adult increased from 21.8% of total calories (1986) to 22.2% (1995) and 23.9% (2003). Adult people who live in rural area consumed less fat than urban (22.9 versus 26.9% of total calories). The problem in our study due to high dietary fat intake rather than consumption of Thai food. Since our study was conducted on a small group of 71 participants who lived in Bangkok, the capital city of Thailand, where they can buy such fast food easily. Thai diet contains some medicinal, low caloric food additives and condiments for being hot and spicy such as garlic, chillies, lime juice, and lemon grass. These not only contribute to distinctive textures and flavors, but also add fiber, vitamins, and minerals, also essential for being a healthy diet. According to studies reviewed in “The New York Times” the spiciness of Thai cuisine has been associated with increased satiety, decreased total caloric intake at a meal, and small boosts in metabolism [20].

These emphasized on the importance of NCEP step I dietary program and the composition of macronutrient together with fat quality and quantity in Thai food for consumers. Three-day food diaries, used to track dietary intake, showed no significant difference of macronutrient intake throughout the study.

Our study provides valuable data regarding egg ingestion in hyperlipidemic adults with Thai food, without medication, showing that even only one egg ingestion significantly increased serum TC and LDL-C levels and the three eggs consumption as well. Our study showed no significant change of serum TG and HDL-C levels after 1 egg or 3 eggs consumption daily for 4 wk when compared to egg washout period. TC/HDL-C ratio may be associated with risk factors for ischemic heart disease or atherosclerosis. Thus the best ratio according to the American Heart Association would be 2 or 3 or less than 4 [21]. The TC/HDL-C ratio in our study was between 3.70 and 3.85. The current National Cholesterol Education Program Adult Treatment Panel III guidelines recommend specific target levels of LDL cholesterol (LDL-C) and HDL cholesterol (HDL-C) for determining cardiovascular disease (CVD) risk [22] which should be between 2.3 and 4.9. The LDL-C/HDL-C ratio in our study was between 2.35 to 2.48. Both TC/HDL-C and LDL-C/HDL-C ratios during the study showed no significant change and remained within normal limit.

From our study results, we would like to remind ones to be careful about eating even 1 egg per day in order to be aware of the amount and composition of fat in dietary intake especially saturated fat and cholesterol intake. The recommendation of NCEP is still important in the treatment of hyperlipidemia, if you strictly follow the recommendation you can have daily consumption of up to 3 eggs without adding on your serum cholesterol level.

## 5. Conclusion

Our study demonstrated that consumption of 1 up to 3 eggs daily was likely to have substantial impact on serum TC and LDL-C levels. We emphasized the importance of the cholesterol lowering diet (NCEP step I diet) to lower both serum TC and LDL-C levels during egg supplement. So, daily egg supplementation together with limited fat intake, especially saturated fat and cholesterol intake is likely to provide a great impact on serum lipid levels. 

## Figures and Tables

**Table 1:** Characteristics of the study population.

Parameters	Baseline	EGG 0	EGG 1	EGG 3
Sex, female : male	63 : 8			
Age (year)	50.79 ± 13.23			
Body weight	57.7 ± 10.5	57.78 ± 10.35	57.86 ± 10.41	57.99 ± 10.55
BMI	23.02 ± 3.9	22.96 ± 3.88	23.07 ± 3.89	23.13 ± 3.93
BMR	1346.27 ± 263.53	1341.86 ± 249.64	1331.37 ± 278.23	1332.45 ± 281.73
Body fat, %	25.08 ± 8.51	25.18 ± 7.48	26.08 ± 9.05	26.48 ± 9.69
Waist	77.3 ± 9.37	78.30 ± 9.04	78.45 ± 9.43	78.61 ± 9.51
Hip	92.63 ± 12.08	93.59 ± 8.26	94.59 ± 7.48	95.03 ± 7.38
WHR	0.93 ± 0.88	0.89 ± 0.51	0.83 ± 0.06	0.83 ± 0.06
Systolic BP	112.87 ± 13.07	111.03 ± 11.83	109.04 ± 11.91	110.31 ± 10.28
Diastolic BP	70.92 ± 7.76	69.99 ± 7.29	68.14 ± 8.19	68.70 ± 7.35
Body cell mass, %	40.49 ± 14.03	40.83 ± 11.98	38.94 ± 12.64	38.79 ± 12.87
Lean body mass, %	74.92 ± 8.51	74.82 ± 7.49	73.92 ± 9.05	73.52 ± 9.69
Total body water, Lt	32.43 ± 7.12	32.28 ± 6.25	32.18 ± 7.35	32.28 ± 7.51

No statistical difference.

**Table 2:** Serum lipid levels of the participants on egg study.

Parameter	Baseline	EGG 0	EGG 1	EGG 3
TG	97.27 ± 56.10	95.64 ± 45.65	97.31 ± 70.15	99.06 ± 57.15
TC	210.96 ± 41.20^a^	212.18 ± 41.56^a^	221.54 ± 42.54^b^	225.31 ± 45.06^b^
LDL-C	133.21 ± 36.26^a^	133.67 ± 36.08^a^	141.38 ± 38.23^b^	145.48 ± 39.33^b^
HDL-C	58.30 ± 12.01^a^	59.39 ± 14.34^a,b^	60.69 ± 12.20^b^	60.01 ± 12.56^a,b^
TC/HDL-C	3.72 ± 0.89	3.70 ± 0.91	3.75 ± 0.87	3.85 ± 0.85
LDL-C/HDL-C	2.36 ± 0.75	2.35 ± 0.80	2.40 ± 0.76	2.48 ± 0.72

^a^Significantly different from ^b^within the same row.
